# Effect of Ghrelin Hormone as a Diagnostic Factor on Appendicitis

**DOI:** 10.1155/2020/4073059

**Published:** 2020-07-27

**Authors:** Ahmad Amouzeshi, Asghar Zarban, Seyede Narjes Yahooian, Bibi Fatemeh Shakhs Emampour

**Affiliations:** ^1^Department of Surgery, School of Medicine, Cardiovascular Diseases Research Center, Birjand University of Medical Sciences, Iran; ^2^Department of Clinical Biochemistry, School of Medicine, Cardiovascular Diseases Research Center, Birjand University of Medical Sciences, Iran; ^3^School of Medicine, Birjand University of Medical Sciences, Iran; ^4^Department of Anesthesiology, School of Medicine, Cardiovascular Diseases Research Center, Birjand University of Medical Sciences, Iran

## Abstract

**Background:**

Appendicitis is the most common cause of surgery in people under 50. In America, it causes 250,000 cases per year and about 1 million days of hospitalization.

**Methods:**

This cross-sectional study was performed on 20 patients under appendectomy with diagnosis of acute appendicitis. The statistical population was divided into two groups, proven appendicitis in pathology and rejection of appendicitis in pathology. Then, 10 patients were assigned into each group.

**Results:**

A total of 20 patients were enrolled in this study, 9 of whom were female (45%) and 11 male (55%). The most common symptom was anorexia. However, there was no statistical difference between the two groups. The lowest level of serum ghrelin was 0.95 and the highest was 16.00 in the study group, which was the mean in people with appendicitis and nonappendicitis 6.24 ± 4.09 and 5.12 ± 4.85, respectively. These values were not significantly different between the two groups (*T* = 0.45, df = 18, *p* = 0.65).

**Conclusion:**

This conclusion may be due to the small number of cases introduced into the study, which suggests that further investigation is warranted with a larger sample size.

## 1. Introduction

Appendicitis is a common disease in the United States causing 250,000 cases per year and approximately 1 million hospitalizations per day [1]. It is the most common cause of surgery in people under 50 and has the highest peak in the 20s and 30s. Women have a higher risk of misdiagnosis and a higher rate of negative appendectomy. Uncommon manifestations are very common in the absence of diagnosis, which increases the risk of mortality and morbidity [2]. The diagnosis is based on 3 factors: history of disease (shift of pain), clinical findings (level of peritoneal irritability), and laboratory tests (inflammatory phase response factors) [3].

The anatomical variation of the location of the inflamed appendix can lead to erroneous examination findings. In retrocecal appendicitis, the anterior abdominal examination has fewer findings, and the presence of tenderness may be more pronounced on the patient's side (Flank). There may be no abdominal findings at all when the inflamed appendix is inside the pelvis. In this case, the diagnosis may be wrong unless rectal examination is done. Although CRP is an indicator of complication appendicitis, the body's inflammatory response to acute appendicitis is a dynamic process. In the early stages of this inflammatory process, the inflammatory responses are weak, as far as the increase in CRP can take up to 12 hours from the start of the process. Rapid diagnosis may minimize the risk of perforation and prevent complications. However, no single symptom, sign, or diagnostic test accurately confirms the diagnosis of appendicitis representing all cases.

Ghrelin, or appetite hormone, known since 1999, is released by the 28-amino acid structure of ghrelin cells in the gastrointestinal tract whose function in the hypothalamic segment increases the desire to eat in humans [4]. This hormone can play a central role in inflammatory and immune processes in many surgical operations [5]. There have also been ascending and descending levels of preoperative and 72 hours postoperative interventions in many abdominal and surgical operations [6]. Given the inflammatory course of appendicitis and the function of ghrelin in inflammatory phases, it may be helpful to diagnose cases of acute appendicitis based on changes in the level of biologic fluids. Some studies investigated ghrelin levels pre and postoperation and showed a significant decrease in ghrelin level in appendectomy surgery [7]. In this study, we examined whether this appetite hormone can help in diagnosis of appendicitis.

## 2. Methods

Patients admitted to the Birjand Imam Reza Hospital with the diagnosis of acute appendicitis undergoing appendectomy surgery based on the criteria set (being healthy, i.e., no other disease and not taking any medication or supplements) were studied. This study was a clinical trial diagnostic with an entry code in the IRCT system. The work was cross-sectional from December 1, 2016, to April 15, 2018. Based on a study by Cetinkaya and colleagues at the Turkish General Surgery Department, to determine the difference between serum and saliva levels, they evaluated the before and after surgery levels in patients with appendicitis and gallstones. Based on the mean ghrelin level between people with appendicitis and the control group comparison of means in two groups sample size about 1 person,

(1) $$\begin{array}{c} n=\frac{{\left(Z_{1-\left(a/z\right)}+Z_{1-\beta }\right)}^{2}\left({S_{1}}^{2}+{S_{2}}^{2}\right)}{{\left(\mu_{1}-\mu_{2}\right)}^{2}}. \end{array}$$

This study used a pilot study of 10 individuals (per group) after selecting the patients according to the established criteria (healthy people not having other diseases and no medication or supplements). Preoperatively, a demographic questionnaire and clinical findings were obtained and according to a questionnaire, a blood sample (5 mm) was taken from the brachial vein in fasting. By centrifuging blood samples, serum was prepared and stored in open-end tubes in freezing conditions at -80°C until testing. The serum levels of ghrelin (ZellBio kit, ELISA) in the samples taken were measured according to manufacturer's instructions. Postoperative appendicitis specimens were placed in pathological vessels and sent to the pathology laboratory. The pathological outcome of the patient was added to the questionnaire information.

Regarding the serum levels of ghrelin in the patients of study, the distribution of data in patients with positive and normal pathology was examined separately using the Shapiro-Wilk test (*p* = 0.002, 0.0001). The results from the test showed that the data did not have a normal distribution. Moreover, serum levels of ghrelin were compared between the two groups using Mann–Whitney test with a significance level of less than 0.05.

## 3. Results

**Figure 1:**
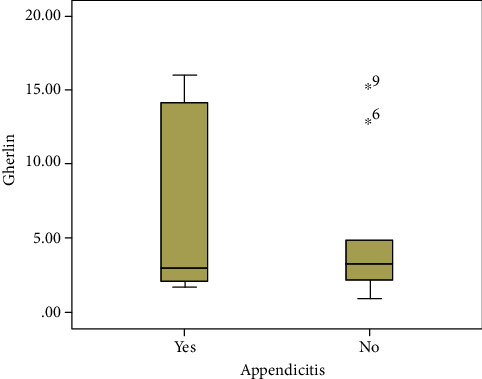
Comparison of preoperative serum ghrelin levels in patients with pathologic diagnosis of appendicitis and without appendicitis.

**Figure 2:**
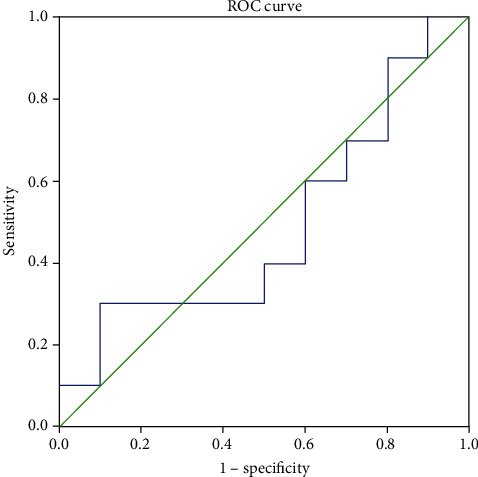
The area under the curve in evaluating the sensitivity and specificity of ghrelin level in the diagnosis of appendicitis.

The demographic feature data and ghrelin level of the two groups are shown in Tables 1 and 2. In patients with pathological diagnosis of appendicitis, the mean and median ghrelin serum levels were 5.69 ± 5.39 (ng/mL) and 3.08 (ng/mL), respectively. However, in those without appendicitis, the mean and median ghrelin serum levels were 5.12 ± 4.85 (ng/mL) and 3.28 (ng/mL). Although patients with appendicitis pathology had higher mean serum ghrelin levels than patients with normal pathology, there was no statistically significant difference between the two groups (Figure 1 and Table 2). The area under the curve in evaluating the sensitivity and specificity of ghrelin level in the diagnosis of appendicitis was 0.500, suggesting it as a poor marker (Figure 2).

## 4. Discussion

Appendicitis is one of the most common causes of abdominal pain requiring surgery, often leading to a difficult diagnosis. The classic signs and symptoms of appendicitis do not always appear. Different symptoms can complicate the diagnosis of acute appendicitis [1]. Given the importance of this topic, this study is aimed at evaluating the effect of ghrelin as a diagnostic factor on preoperative appendicitis. In this study, it was concluded that although ghrelin was higher in patients with appendicitis than in nonappendicitis patients, this value was not statistically significant.

Ghrelin is secreted as an anorexic hormone from the stomach. Given this characteristic and anorexia symptom in appendicitis patients as a common symptom, it is expected that an increase in this hormone would indicate the occurrence of appendicitis. In a study by Cetinkaya et al. to determine the difference between serum and salivary levels before and after surgery in patients with appendicitis and gallstone, 2 ml of saliva and 9 ml of blood were obtained from 20 appendicitis patients, 10 cholecystitis patients, and 16 healthy individuals. Preoperative serum ghrelin levels in saliva and serum were significantly lower than the postoperative levels in both patients undergoing appendectomy and controls. They concluded that low serum ghrelin levels in patients with acute appendicitis help in the diagnosis of the disease [7]. Ghrelin levels have also been evaluated in other surgical procedures. In a study by Kontoravdis et al., serum levels of ghrelin after colectomy and cholecystectomy were significantly higher compared to preoperation [5]. In the study by Maruna et al., the results of the Kontoravdis study also confirmed the effects of stress-induced action on changes in serum ghrelin levels. Remarkably, after 48 hours, ghrelin levels returned to preoperative levels [6].

The lack of such a finding in the present study could be attributed to the significant frequency of anorexia in both groups of patients with appendicitis pathology and normal pathology. Consistent with the results of this study, previous studies have also shown that anorexia is highly prevalent in patients with suspected appendicitis and, despite its high sensitivity, has very low specificity [8]. In this study, it was also suggested that although anorexia increases the likelihood of appendicitis, its absence cannot play a role in the diagnosis of acute appendicitis. Overall, it was observed that anorexia is not a highly specific factor in the diagnosis of appendicitis. On the other hand, according to the results of this study, this hormone has a low specificity in the diagnosis of appendicitis, yet research with a larger sample size is recommended.

## Figures and Tables

**Table 1:** Comparison of preoperative serum ghrelin levels in patients with pathologic diagnosis of appendicitis and without appendicitis.

Pathologic	Ghrelin level (ng/ml)	Statistical test result
Appendicitis	5.69 ± 5.39	∗*p* = 1
Normal	5.12 ± 4.85	

^∗^Results of Mann–Whitney *U* test revealed no significant difference between the two groups.

**Table 2:** Comparison of gender and age in patients' appendicitis.

Gender	Frequency	Percentage
Females	9	45
Males	11	55
Total	20	100
Age ≤ 30 (year)	12	60
Age > 30 (year)	8	40
Total	20	100

## Data Availability

The data used to support the findings of this study are available from the corresponding author upon request.

## Additional Points

*Limitations.* One of the limitations of this study was the partly low size and also the significant frequency of anorexia in suspicious patients (with normal pathology). Researchers recommend that these two issues be considered in future studies.
